# An Empirical Bayesian Method for Detecting Differentially Expressed Genes Using EST Data

**DOI:** 10.1155/2008/817210

**Published:** 2008-03-13

**Authors:** Na You, Junmei Liu, Chang Xuan Mao

**Affiliations:** Department of Statistics, University of California, Riverside, CA 92521, USA

## Abstract

Detection of differentially expressed genes from expressed sequence tags (ESTs) data has received much attention. An empirical Bayesian method is introduced in which gene expression patterns are estimated and used to define detection statistics. Significantly differentially expressed genes can be declared given detection statistics. Simulation is done to evaluate the performance of proposed method. Two real applications are studied.

## 1. INTRODUCTION

It is important to detect differentially expressed genes, for example, exploring the key genes related to certain diseases. As the EST sequencing technology develops, a large number of EST databases from a variety of tissues are available. Enormous EST collections provide opportunities to quantify gene expression levels [1]. Efficient statistical methods are in great demand.

Several methods have been proposed to detect significantly differentially expressed (SDE) genes from EST data [2]. Fisher's exact test was used by the Cancer Genome Anatomy Project [3]. Audic and Claverie [4] developed a Bayesian method. GT statistic [5] and R statistic [6] were proposed for multilibrary comparison. In each method, gene-specific detection statistics quantify differences of gene expression levels and SDE genes are declared by their rankings.

An empirical Bayesian method is proposed to detect SDE genes. The relative gene expression abundances are estimated in each library, and a new detection statistic is derived for each gene. In Section 2, simulation experiments suggest that the proposed method outperforms those existing methods. Real applications are also studied in Section 2. Statistical methods are described in Section 3. The possibility of extending the method for multiple libraries is indicated in Section 4.

## 2. RESULTS

Let (*π*_11_, *π*_12_,…, *π*_1*c*_) and (*π*_21_, *π*_22_,…, *π*_2*c*_) be the gene expression patterns in two libraries, where *π*_*ji*_ is the relative abundance of gene *i* in library *j*. The absolute difference between relative abundances is *D*_*i*_ = |*π*_1*i*_ − *π*_2*i*_|. Given a sample of ESTs from library *j*, an empirical Bayes estimator π^ji for *π*_*ji*_ is defined in Section 3. Given gene *i* seen in both samples, define ${\hat{D}}_{i}=|{\hat{\pi }}_{1i}-{\hat{\pi }}_{2i}|$. Given gene *i* seen in only one sample, for example, sample 2, define ${\hat{D}}_{i}=|{\hat{\pi }}_{1i}-{\hat{\pi }}_{2i}|$ if ${\hat{\pi }}_{1i}<{\hat{\pi }}_{2i}$ and ${\hat{D}}_{i}=0$ otherwise, which is conservative in the sense that ${\hat{D}}_{i}$ possibly underestimates *D*_*i*_. Gene *i* is declared to be SDE if ${\hat{D}}_{i}$ is relatively large.

### 2.1. Simulation

In a simulation experiment, EST frequencies are generated from a multinomial distribution with sample size *s*_*j*_ and probability vector (*π*_*j*1_, *π*_*j*2_,…, *π*_*jc*_), where *c* = 1000, *π*_*ji*_ = *λ*_*ji*_/∑^*c*^_*k*=1_ *λ*_*jk*_, (*λ*_11_, *λ*_12_,…, *λ*_1*c*_) from *G*_1_, (*λ*_21_, *λ*_22_,…, *λ*_2*c*_) from *G*_2_, and *G*_1_ and *G*_2_ are two distributions over (0, *∞*). The proposed methods, Fihser's exact test, *χ* ^2^ test, AC statistic, and R statistic, are studied. Given a cutoff point *τ*, the efficiency of a statistical method is measured by *p*_*τ*_, the expected percentage of the true first *τ* SDE genes being correctly declared as the first *τ* SDE genes. The average of estimated *p*_*τ*_ is calculated from 500 replications.

**Figure 1:**
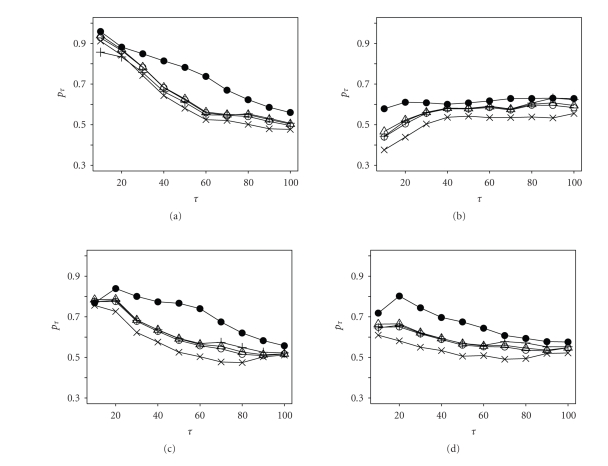
Simulation results of Fisher's exact test (∘), *χ* ^2^ test (Δ), AC statistic (+), R statistic (×), and the proposed statistic (•) in detecting SDE genes using two EST samples of the same size.

In the first four experiments, *s*_1_ = *s*_2_ = 2000 and the results are presented in Figure 1. Note that *G*_1_ = *U*(0, 10), Beta(2, 5), 0.2*δ*(2) + 0.4*δ*(5), 0.2*δ*(10) Gamma(3, 0.1) and *G*_2_ = Beta(2, 1), Beta(2, 5), Beta(2, 2), Beta(2, 2), respectively,where *U*(*a*, *b*) is the uniform distribution on (*a*, *b*), *δ*(*a*) is degenerate at *a*, Beta(*a*, *b*) is transformed from the beta distribution with shape parameters *a* and *b* by *λ* = *p*/(1 − *p*) for *p* ∈ (0, 1), and Gamma(*a*, *b*) is the gamma distribution with shape *a* and scale *b*. For each cutoff point *τ* = 10, 20,…, 100, *p*_*τ*_ are calculated. Clearly the proposed method has better performance than others.

**Figure 2:**
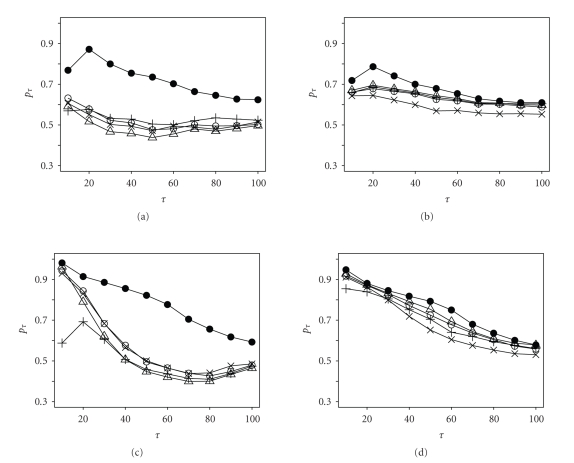
Simulation results of Fisher's exact test (∘), *χ* ^2^ test (Δ), AC statistic (+), R statistic (×), and the proposed statistic (•) in detecting SDE genes using two EST samples of different sizes.

In the second four experiments, (*s*_1_, *s*_2_) = (2000, 4000), (4000, 2000), (2000, 4000), and (4000, 2000) respectively, and the results are presented in Figure 2. Note that *G*_1_ = Gamma(3, 0.1) and *G*_2_ = Beta(2, 2) in Figures 2(a) and 2(b) and *G*_1_ = *U*(0, 10) and *G*_2_ = Beta(2, 1) in Figures 2(c) and 2(d). The proposed method is usually the best one among all methods studied.

### 2.2. Real applications

One example concerns Chinese spring wheat drought stressed leaf cDNA library (7235) and root cDNA library (#ASP), available at TIGR gene indexes database (downloaded at http://www.tigr.org/tdb/tgi, 01/06/2006). In each EST sample, there are totally 790 and 1306 sequenced ESTs, respectively. After removing the unannotated 103 and 194 ESTs, the annotated ESTs are clustered into 465 and 804 groups with each group associated with a unique gene. Only those well-annotated ESTs are used. The first 20 SDE genes by the proposed method are listed in Table 1, among which 7, 7, 7, and 7 genes are in the set of first 20 SDE genes by Fisher's exact test, *χ* ^2^ test, AC statistic, and R statistic, respectively.

Another example concerns pinus gene expression level comparison in root gravitropism April 2003 test library (#FH3) and root control 2 (late) library (#FH4), also from TIGR, in which 2513 and 1132 ESTs associated with 1211 and 605 genes are well annotated and clustered. Table 2 lists the first 20 SDE genes by the proposed method, among which 4, 4, 5, and 3 genes are in the set of the first 20 SDE genes by Fisher's exact test, *χ* ^2^ test, AC statistic, and R statistic, respectively.

## 3. METHODS

Suppose that there are *c* genes in a library. Let *x*_*i*_ be the number of ESTs from gene *i*, a Poisson variable with mean *λ*_*i*_. Given a prior distribution *G* on the *λ*_*i*_, the posterior mean of *λ*_*i*_ is *E*(*λ*_*i*_ ∣ *x*_*i*_) = (*x*_*i*_ + 1)*h*_*G*_(*x*_*i*_ + 1)/*h*_*G*_(*x*_*i*_), where *h*_*G*_(*x*) = ∫ *λ* ^*x*^/*x*!*e* ^−*λ*^ *d* *G*(*λ*) is a Poisson mixture. A gene is observed if and only if *x*_*i*_ ≥ 1. Conditioning on *x*_*i*_ ≥ 1, *x*_*i*_ follows a zero-truncated Poisson mixture *h*_*G*_(*x*)/(1 − *h*_*G*_(0)) or a mixture *f*_*Q*_(*x*) of truncated Poisson, where

(1) $$\begin{array}{ll} f_{Q}(x) & =\frac{h_{G}(x)}{1-h_{G}(0)}=\int \frac{\lambda^{x}}{x!(e^{\lambda }-1)}dQ(\lambda ),\\ dQ(\lambda ) & =\frac{\left(1-e^{-\lambda })dG(\lambda \right)}{\int (1-e^{-\eta })dG(\eta )}. \end{array}$$

Let *θ*(*Q*) = *h*_*G*_(0)/(1 − *h*_*G*_(0)) be the odds that a gene is unseen. Write *E*(*λ*_*i*_ ∣ *x*_*i*_) = *f*_*Q*_(1)/*θ*(*Q*) if *x*_*i*_ = 0 and *E*(*λ*_*i*_ ∣ *x*_*i*_) = (*x*_*i*_ + 1) *f*_*Q*_(*x*_*i*_ + 1)/*f*_*Q*_(*x*_*i*_) otherwise.

Let *n*_*x*_ denote the number of genes with exactly *x* ESTs in the sample. The nonparametric maximum likelihood estimator $\hat{Q}$ for *Q* is

(2) $$\hat{Q}=\text{argmax}\sum_{x\geq 1}n_{x}\log f_{Q}(x),$$

whose calculation is discussed in [7]. It is difficult to estimate *θ*(*Q*) well [8]. There are lower bound estimators, for example, $\hat{\theta (Q)}=n_{1}(n_{1}-1)/\{2n(n_{2}+1)\}$ [9], where *n* = ∑_*x*≥1_ *n*_*x*_ is the number of observed expressed genes. An empirical Bayes estimator for *λ*_*i*_ is

(3) λ^i=E(λi∣xi)^={fQ^(1)θ(Q)^,xi=0,(xi+1)fQ^(xi+1)fQ^(xi),xi≥1.

As the relative abundance *π*_*i*_satisfies *π*_*i*_ = *λ*_*i*_/∑^*c*^_*k*=1_ *λ*_*k*_, let ${\hat{\pi }}_{i}={\hat{\lambda }}_{i}/\hat{s}$, where

(4) s^=∑k=1c^λ^k=nfQ^(1)+∑x≥1nx(x+1)fQ^(x+1)fQ^(x),            c^=n{1+θ(Q)^}.

## 4. DISCUSSION

A new statistical method is proposed to compare the gene expression patterns in two cDNA libraries. It can be extended to multilibrary comparison, for example, considering all pairwise comparisons among multiple libraries [3].

## Figures and Tables

**Table 1:** The first 20 SDE genes in wheat leaf and root libraries by the proposed method (*x*_1*i*_-the EST number of gene *i* from leaf library, *x*_2*i*_-that from root library, 0/1-absence/presence in the set of the first 20 SDE genes).

Gene	*x*_1*i*_	*x*_2*i*_	1000*D*_*i*_	Fisher	*χ* ^2^	AC	R
TC24953	19	0	27.10	1	1	1	1
TC23443	8	2	7.88	1	1	1	1
TC23215	1	8	4.87	0	0	0	0
TC26419	1	8	4.87	0	0	0	0
TC26431	1	8	4.87	0	0	0	0
TC24980	5	0	3.40	1	1	1	1
TC23786	0	6	2.62	0	0	0	0
TC26436	0	6	2.62	0	0	0	0
TC24819	0	6	2.62	0	0	0	0
TC26455	7	12	1.85	0	0	0	0
TC23314	1	5	1.59	0	0	0	0
TC24981	1	5	1.59	0	0	0	0
TC24795	0	5	1.57	0	0	0	0
TC24804	0	5	1.57	0	0	0	0
TC26553	0	5	1.57	0	0	0	0
TC26356	4	1	1.37	0	0	0	0
TC23560	4	0	1.37	1	1	1	1
TC24669	4	0	1.37	1	1	1	1
TC24679	4	0	1.37	1	1	1	1
TC26379	4	0	1.37	1	1	1	1

**Table 2:** The first 20 SDE genes in #FH3 and #FH4 by the proposed method (*x*_1*i*_-the EST number of gene *i* from #FH3, *x*_2*i*_-that from #FH4, 0/1-absence/presence in the set of the first 20 SDE genes).

Gene	*x*_1*i*_	*x*_2*i*_	1000*D*_*i*_	Fisher	*χ* ^2^	AC	R
TC40351	4	9	7.62	1	1	1	1
TC40355	6	10	6.25	1	1	1	0
TC51779	19	2	5.12	0	0	1	0
TC40566	7	7	4.03	0	0	0	0
TC51682	7	7	4.03	0	0	0	0
TC46290	14	2	3.79	0	0	0	0
TC46372	15	5	3.70	0	0	0	0
TC40768	13	3	3.40	0	0	0	0
TC40912	13	4	3.36	0	0	0	0
TC51995	12	3	2.94	0	0	0	0
TC40420	12	5	2.56	0	0	0	0
TC51708	18	12	2.44	0	0	0	0
TC40405	11	4	2.43	0	0	0	0
TC40361	0	6	2.34	1	1	1	1
TC46426	0	6	2.34	1	1	1	1
TC46276	19	12	2.12	0	0	0	0
TC40388	9	1	1.82	0	0	0	0
TC40647	9	2	1.82	0	0	0	0
TC40350	9	3	1.81	0	0	0	0
TC40731	8	2	1.63	0	0	0	0
